# Downregulation of the Hsp90 System Causes Defects in Muscle Cells of *Caenorhabditis Elegans*

**DOI:** 10.1371/journal.pone.0025485

**Published:** 2011-09-28

**Authors:** Andreas M. Gaiser, Christoph J. O. Kaiser, Veronika Haslbeck, Klaus Richter

**Affiliations:** Department of Chemistry and Center for Integrated Protein Science Munich (CIPSM) and Technische Universität München, München, Germany; University Medical Center Groningen, The Netherlands

## Abstract

The ATP-dependent molecular chaperone Hsp90 is required for the activation of a variety of client proteins involved in various cellular processes. Despite the abundance of known client proteins, functions of Hsp90 in the organismal context are not fully explored. In *Caenorhabditis elegans*, Hsp90 (DAF-21) has been implicated in the regulation of the stress-resistant dauer state, in chemosensing and in gonad formation. In a *C. elegans* strain carrying a DAF-21 mutation with a lower ATP turnover, we observed motility defects. Similarly, a reduction of DAF-21 levels in wild type nematodes leads to reduced motility and induction of the muscular stress response. Furthermore, aggregates of the myosin MYO-3 are visible in muscle cells, if DAF-21 is depleted, implying a role of Hsp90 in the maintenance of muscle cell functionality. Similar defects can also be observed upon knockdown of the Hsp90-cochaperone UNC-45. In life nematodes YFP-DAF-21 localizes to the I-band and the M-line of the muscular ultrastructure, but the protein is not stably attached there. The Hsp90-cofactor UNC-45-CFP contrarily can be found in all bands of the nematode muscle ultrastructure and stably associates with the UNC-54 containing A-band. Thus, despite the physical interaction between DAF-21 and UNC-45, apparently the two proteins are not always localized to the same muscular structures. While UNC-45 can stably bind to myofilaments in the muscular ultrastructure, Hsp90 (DAF-21) appears to participate in the maintenance of muscle structures as a transiently associated diffusible factor.

## Introduction

The dimeric molecular chaperone Hsp90 is an ATP-dependent cellular machine, which contributes to the activation and regulation of steroid receptors, protein kinases and several transcription factors [1]–[4]. In order to process client proteins, Hsp90 has to hydrolyze ATP in a cyclic reaction. ATP hydrolysis induces conformational changes in the dimeric chaperone, which rearrange domains and thus influence conformations within the substrate proteins, activating and stabilizing them [5]–[7]. Inhibition of Hsp90 by specific compounds, like radicicol or geldanamycin, leads to a fast degradation of many Hsp90 clients [8], [9].

Hsp90 has been studied *in vivo* in vastly different organisms: While bacterial Hsp90 is not essential [10], Hsp90 is an indispensable protein in yeast and in all other eukaryotes studied to date [11]. This might be due to the need to activate and stabilize essential client proteins in an Hsp90 dependent reaction [12]. In the metazoan model system *Caenorhabditis elegans* the Hsp90-homolog DAF-21 is critical for gonad and vulva development as well as oocyte maturation [13]–[15]. Extensive studies in *Drosophila melanogaster* illustrate that the inhibition of Hsp90 uncovers a wealth of phenotypes, including deformed legs and eyes and abnormalities in wings, thorax and bristles [16]. In vertebrates, the disruption of Hsp90's function leads to lethal damage during early development [17], [18]. In vertebrates Hsp90 has also been implicated in the proper formation of muscle structures. Specifically, an Hsp90a.1 knockdown in zebrafish embryos affects the formation of the myofibrillar ultrastructure and it is suspected that Hsp90 participates in these events together with its cofactor Unc-45 [19]. Unc-45, besides binding Hsp90 via its TPR-domain, contains a myosin II interacting UCS domain, and is thought to facilitate the folding process of the myosin head domain. In nematodes, the Hsc70/Hsp90-interacting protein UNC-45 was found to be associated with muscle thick filaments and required for muscle development as well [20]–[22]. Downregulation leads to paralysis and a defective thick filament assembly [23]. Failure to assemble thick filaments and irregular sarcomeric spacing is also observed in fruit flies upon Unc-45 depletion [24] and even overexpression of Unc-45b in zebrafish embryos showed effects on myosin thick filament organization [25].

Here, we analyze the involvement of DAF-21 in muscle function in the nematode *Caenorhabditis elegans*. The effects of a particular point mutation (E292K-DAF-21) in the coding sequence of DAF-21 have been shown before in the nematode strain JT6130 (*daf-21(p673)*) [26]. Individuals of this strain enter the stress-resistant dauer state with a high frequency and rest in this state for several days before proceeding with development [26], [27]. We observe a high incidence of premature mortality in this strain and a generally reduced motility. We further find abnormalities in the muscular tissue upon knockdown of DAF-21, which imply the participation of this molecular chaperone during the maintenance of the muscular ultrastructure in nematodes.

## Results

### daf-21(p673) nematodes are characterized by high mortality and motility defects

**Figure 1:**
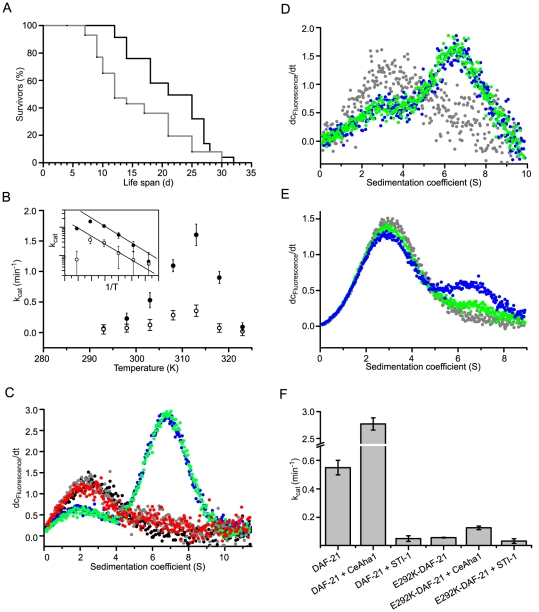
*daf-21(p673)* nematodes show a shortened adult life time and contain a compromised version of DAF-21. A. A Kaplan-Meier plot comparing N2 (black line) and JT6130 (grey line). 80 individuals of each strain were subjected to lifespan analysis starting with freshly hatched L1 larvae in 5 separate experiments. The worms were was kept at 20°C. Day 1 of the experiment is the hatching day of synchronized larvae. The mean lifespan was 18.7 d±1.7 d for N2 and 12.8 d±1.8 d for JT6130. B. ATPase activities of DAF-21 (filled circles) or E292K-DAF-21 (open circles) were determined at varying temperature. The assays were performed in 40 mM HEPES/KOH, pH 7.5, 80 mM KCl at the indicated temperatures and evaluated according to the Materials and Methods section. The inset in the upper left corner shows the Arrhenius plot of the same data set. The data points show a linear relationship up to roughly 40°C. C. The functionality of DAF-21 is confirmed by its ability to bind the cofactor Cep23, which specifically recognizes the hydrolysis competent state with closed N-terminal domains. Labelled Cep23 alone sedimented with an s value of 2.3 S (black circles). The sedimentation was not affected by the presence of DAF-21 or E292K-DAF-21 (red and grey circles). If additional 4 mM AMP-PNP was present, the formation of a complex was observed at 6.8 S for both, DAF-21 (blue circles) and E292K-DAF-21 (green circles). D. Binding of Aha1 to DAF-21 and E292K-DAF-21 was measured by using labelled CeAha1. CeAha1 alone sedimented with an s value of 3.4 S (grey circles). Complex formation with DAF-21 or E292K-DAF-21 could be observed at 6.7 S (blue and green circles, respectively). Both variants showed almost identical complex binding to CeAha1. E. Binding of STI-1 to DAF-21 and E292K-DAF-21 was measured by using labelled STI-1. STI-1 alone sedimented at 3.0 S (grey circles). Addition of DAF-21 (blue circles) or E292K-DAF-21 (green circles) led to the formation of a 6.5 S complex. Complex formation was weaker for E292K-DAF-21 suggesting that the binding to the E292K-DAF-21 variant is reduced compared to DAF-21. F. The ATPase activities of DAF-21 and E292K-DAF-21 are influenced by the cofactors CeAha1 and STI-1. DAF-21 is strongly activated by CeAha1 and strongly inhibited by STI-1; the effects on E292K-DAF-21 are weaker. The ATPase activities were measured at 30°C in a low salt buffer as indicated in the Materials and Methods section.

The nematode strain JT6130 (*daf-21(p673)*) is characterized by the single point mutation E292K in the open reading frame of *daf-21* [26]. We investigated the influence of this mutation on the lifespan of the nematode. Synchronized wild type (wt) worms of the N2 strain and *daf-21(p673)* nematodes were subjected to lifespan assays. For JT6130 only individuals that did not enter the dauer state and thus reached adulthood within the same day as the N2 worms were evaluated. About 50% of the JT6130 nematodes died between days 8 and 10 from bagging, while the remaining 50% did not show a reduced lifespan compared to N2 nematodes (Figure 1A). Throughout their life, *daf-21(p673)* nematodes showed a reduced motility compared to N2 worms according to thrashing assays at different time points during the lifespan experiment (Table 1). The amount of bends in a defined time period was already reduced by about 30% at the beginning of adulthood and remained lower throughout the aging process. In order to confirm the involvement of DAF-21 in the observed phenotypes, we reduced DAF-21 levels in wt worms by feeding RNA interference (RNAi) using a construct described before [14], [28]. Worms remained sterile as previously observed [13]–[15]. We used worms at day 4 and day 7 after hatching and measured the frequency of thrashing (Table 1). In comparison to control worms, movements are markedly reduced, confirming a role of DAF-21 in processes associated to motility.

**Table 1:** Motility of nematodes of different age.

Time after hatching	N2	*daf-21(p673)*	*control* RNAi	*daf-21* RNAi	*unc-45* RNAi
(d)	(Strokes per min ± stdev)				
4	95±6	73±10	85±8	61±12	10±3
7	91±8	55±5	87±9	38±6	4±3
10	94±15	63±6	n.d.	n.d.	n.d.

The motility of the wt strain N2, the strain JT6130 (*daf-21(p673)*) and N2 depleted for DAF-21 or UNC-45 were determined as described in the Materials and Methods section at the indicated time points during aging.

### The E292K-DAF-21 variant is a stable protein with compromised functionality

*Daf-21* is an essential gene, as observed by RNAi and knockout strains [15]. Given the essential nature of DAF-21, we were intrigued by the mild phenotype of the *daf-21(p673)* nematodes. In order to understand the impact of the E292K mutation on the functionality of the protein, we analyzed purified E292K-DAF-21 biochemically. At 30°C, the ATPase activity of E292K-DAF-21 was found to be 0.1 min^−1^, while that of wt-DAF-21 was 0.6 min^−1^ (Figure 1B). We were interested, whether the reduced activity is coupled to an intrinsic destabilization of the mutated protein. Hence, we analyzed the activity of the wt and variant proteins at different temperatures. We observed an increase of the activity up to 40°C, while higher temperatures resulted in a loss of activity for both proteins (Figure 1B). Between 20°C and 25°C this effect is subtle. Converting the data into an Arrhenius plot (inset of Figure 1B), the linear increase in log(k_cat_) for both proteins becomes more pronounced and reaches close to 40°C, suggesting that the enzymatic activity of both proteins is stable at temperatures well above the normal growth temperature of nematodes. Urea induced unfolding transitions confirm a similar structural stability, as the transition curves for both proteins are basically superimposable (Figure S1). Thus, the E292K-mutation does not result in a destabilization of the protein but rather reduces the intrinsic activity of DAF-21. Given the mutation exchanges a charged residue to another charged residue on the protein surface, a structural destabilization should not be expected (Figure S2). Additionally we found the chaperone activity of the protein to be unaffected by the mutation (Figure S3).

As E292K-DAF-21 has a lower ATPase activity, we aimed at understanding, whether the binding to known Hsp90-cofactors and its ability to perform conformational changes are affected by the mutation. We thus labelled the cofactors Cep23 (ZC395.10), CeAha1 (C01G10.8) and STI-1 with fluorescein derivates and subjected them to analytical ultracentrifugation in the absence and presence of DAF-21 or the E292K variant. As described in the literature [29], a complex of DAF-21 and Cep23 forms in the presence of the nucleotide AMP-PNP, but is undetectable in the absence of this nucleotide (Figure 1C). The variant as well as the wt protein can form this complex equally well, suggesting that the nucleotide induced conformational changes can still be performed despite the lower ATPase activity. Similarly, the binding to the cofactor CeAha1 is not affected by the mutation (Figure 1D). Surprisingly, the binding to the cofactor STI-1 is slightly compromised, as a reduced formation of the 6.7 S STI-1-DAF-21 complex can be observed (Figure 1E). To confirm that binding of cofactors still leads to the expected effects on the ATPase activity we tested, whether CeAha1 can stimulate the ATPase activity of the mutated protein. Indeed, addition of CeAha1 to E292K-DAF-21 resulted in an increase of the ATPase activity, although this increase was much smaller than for DAF-21 (Figure 1F). Also, the inhibition of its already very low activity by the cofactor STI-1 could be confirmed (Figure 1F). Thus, despite a reduced turnover rate, the interaction of E292K-DAF-21 with its cofactors is still possible and in principle functional. This slightly compromised functionality may explain the mild effects of the mutation *in vivo*, compared to the larval arrest phenotype of knockout strains [15], [26], [30] and the sterility upon *daf-21* knockdown. Apparently, the most essential activities of the DAF-21 machinery are still supported despite the substitution of the glutamate at residue 292 to lysine. In this context it is also important to note, that the ability of the compromised Hsp90 variant to hydrolyze ATP is still well above that of both human Hsp90 homologs [31].

### Daf-21 is ubiquitously expressed even at non-stress conditions

**Figure 2:**
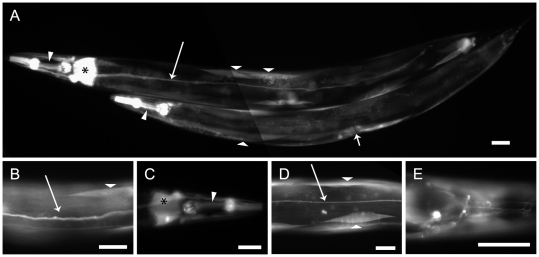
*daf-21* is ubiquitously expressed in *Caenorhabditis elegans*. A–C. Nematodes of the BC10293 strain, which contains a transcriptional fusion of GFP to the *daf-21* promoter are shown. Fluorescence can be detected in the excretory canal cell (long arrow), in the pharyngeal muscle cells (sharp triangle) and the cells of the first intestinal ring (asterisk). Further individual muscle cells (blunt triangle) and the vulval muscles (short arrow) show fluorescence (A). Detailed views of the same strain highlight the excretory canal cell (long arrow), an individual muscle cell (blunt triangle) (B), the pharyngeal muscle cells (sharp triangle) and the cells of the first intestinal ring (asterisk) (C). D–E. Newly generated transgenic nematodes, carrying a *daf-21* promoter sequence fused to YFP. Fluorescence is detected in the excretory canal cell (long arrow) and body wall muscle cells (wide triangles) (D). Fluorescence is also pronounced in some head neurons (E). The scale bar represents 40 µm in all subpanels.

DAF-21 had not been picked up in a recent screen of proteins, which are preferentially expressed in muscle cells [32]. We aimed at defining the tissues expressing *daf-21* in order to understand the motility defect phenotype. We investigated the stable reporter strain BC10293, which contains a transcriptional fusion of GFP to the *daf-21* promoter [33]. Individual nematodes of this strain showed varying expression patterns. The *daf-21* promoter led to GFP expression in the excretory system, pharyngeal muscle cells and intestinal cells (Figures 2A, 2B and 2C). Furthermore, sporadic expression could be observed in body wall muscle cells (Figure 2A and 2B). In order to confirm the sporadic expression in muscles, we constructed another reporter plasmid, containing a 2.5 kb genomic sequence upstream of the DAF-21 start codon including its first 4 amino acids fused to YFP. This plasmid was microinjected into N2 nematodes. We were able to obtain about 100 F1 offspring, some of them brightly fluorescent. No stable line was obtained though, but similar to the stable strain BC10293, F1 showed fluorescence in differing tissues. The fluorescent tissues corresponded to those of the stable line and the sporadic expression in body wall muscle cells was recapitulated (Figure 2D). Additionally, fluorescence in single head neurons was observed (Figure 2E). These data confirm that the Hsp90 protein in nematodes is constitutively expressed in many tissues, including body wall muscle cells.

### DAF-21 is barely heat inducible, but suppresses the muscular heat shock response

**Figure 3:**
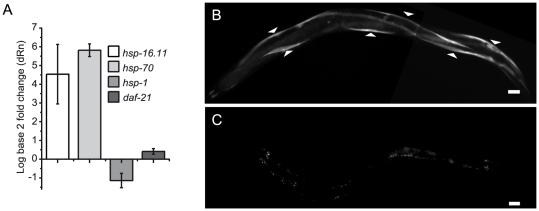
*daf-21* knockdown leads to the induction of the muscular stress response. A. Real-Time Quantitative PCR measurements illustrating the relative abundance of *hsp-16.11*, *hsp-70*, *hsp-1* and *daf-21* before and after heat shock. Relative abundance was determined in relation to the levels of both, *act-1* and *pgk-1* mRNA. The mean and standard deviation comparing four separate biological replicates is given. B–C. Nematodes of the integrated *hsp-70::GFP* reporter strain [28], [39]. Upon feeding RNAi against *daf-21* for three days, these worms exhibited GFP fluorescence in the body wall muscle cells (blunt triangles) (B), whereas untreated age-matched individuals did not exhibit specific fluorescence, despite longer exposure times (C). At these longer exposure times unspecific fluorescence in the intestinal cells is detected. The scale bar represents 20 µm in both micrographs.

Hsp90 is a well described heat shock protein [34]. We aimed at understanding, whether DAF-21 is upregulated in the course of the heat shock response. No increased induction was observed, when individual nematodes of the BC10293 strain were subjected to a heat shock (data not shown). To analyze the transcription level of *daf-21*, we performed real time quantitative PCR measurements on heat shocked and non heat shocked nematodes (Figure 3A). The transcription of *daf-21* was barely affected by heat-stress, although the heat-inducible genes *hsp-70/C12C8.1* and *hsp-16.11/T27E4.2* were upregulated about 40-fold in response to heat-treatment. The constitutively-expressed Hsc70-homolog *hsp-1/F26D10.3* was slightly reduced in response to heat shock (Figure 3A). Thus, *daf-21* is constitutively expressed in various tissues and its expression is not strongly affected by heat shock.

Beyond being a heat shock protein, Hsp90 is known to participate in the regulation of the heat shock response based on studies in several organisms [34], [35]. An inhibition of Hsp90 by small molecules strongly induces Hsp70 and other heat shock proteins in mammalian cell culture [36]–[38]. To test, whether this function is conserved for DAF-21, we deployed a heat shock reporter strain. In this strain, the heat-inducible *hsp-70* promoter controls GFP expression (*hsp-70::GFP*). This strain shows GFP induction in many tissues after heat shock [28], [39]. Interestingly, feeding these *hsp-70::GFP* nematodes with RNAi directed against *daf-21* resulted in the specific appearance of GFP in body wall muscle cells (Figure 3B), while nematodes treated with control RNAi did not show GFP induction (Figure 3C). Thus, upon reduction of Hsp90 levels, in particular the stress response in muscle cells is initiated, leading to the expression of chaperone proteins.

### Knockdown of daf-21 and unc-45 leads to aggregation of myosin in body wall muscle cells

**Figure 4:**
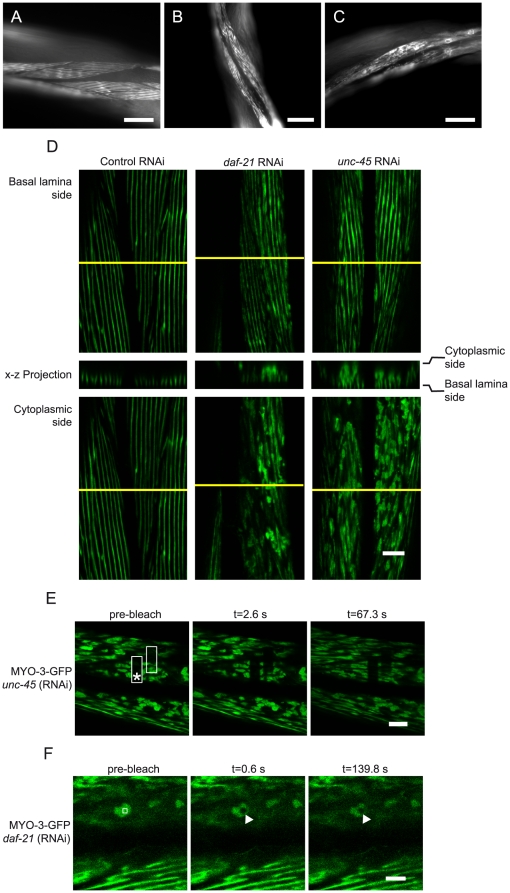
*daf-21* and *unc-45* knockdown leads to an accumulation of aggregate-like myosin in muscle cells. A-C. RNAi experiments were performed in the strain RW1596, which shows details of muscular organization due to the expression of a MYO-3-GFP fusion protein. The worms were treated either with control RNAi (A) or RNAi against *daf-21* (B) or *unc-45* (C). The scale bar represents 40 µm for all micrographs. D. Animals, treated as in A–C were also analyzed by confocal microscopy. Although the striated pattern seems not to be compromised by either knockdown in an optical sectioning plane close to the basal lamina (upper row), in sections closer to the muscle cell body (lower row), aggregates are visible when UNC-45 or DAF-21 are depleted. A projection of the x-z plane along the yellow line in both x-y sections further highlights the accumulation of misplaced MYO-3 at the cytoplasmic side of the sarcomere lattice. The scale bar represents 10 µm and applies to all micrographs in the panel. E. The structures found in animals treated with RNAi against *unc-45* were subjected to FRAP analysis and recovery was recorded for 67 seconds. No recovery is observable. The bleaching site of the previous experiment is still visible after several minutes, as indicated by the box marked with an asterisk. The scale bar represents 10 µm and applies to all micrographs in the panel. F. Similar structures in individuals treated with RNAi against *daf-21* were as well analyzed by FRAP. The bleaching site is marked by a box in the leftmost picture and by a triangle during the subsequent recovery time. The scale bar represents 10 µm in all micrographs of the panel.

Since a compromised DAF-21 functionality results in a phenotype that may be attributed to the muscle system, we used the nematode strain RW1596, which expresses a fusion of GFP to the myosin protein MYO-3 in muscle cells [40] in order to monitor the effect of *daf-21* knockdown on the muscular ultrastructure. Interestingly, whereas muscle striation appeared homogenous and parallel in untreated nematodes of the RW1596 strain (Figure 4A), in worms exposed to *daf-21* RNAi, the muscle structure appeared inhomogeneous and disrupted (Figure 4B). We could observe an even stronger effect upon knockdown of the Hsp70/Hsp90-cofactor *unc-45*, resulting in a dramatic disorganization of the muscle structure (Figure 4C) and almost complete loss of motility (Table 1). While 100% of the animals were affected in response to *unc-45* knockdown at day 5 after synchronization, about 60% of the nematodes showed signs of muscular disorganization in response to *daf-21* RNAi, scoring 40 animals.

In order to obtain a deeper insight into how the muscle cells are affected, we examined the nature of the structural disruption by confocal microscopy. We compared nematodes exposed to *daf-21* RNAi, *unc-45* RNAi and control RNAi. Upon knockdown of *daf-21* and *unc-45*, the striation of the muscle cell seemed to be mostly intact, but fluorescent patches became visible. These patches originate from the localization of GFP-MYO-3 to the muscle cell body outside of the sarcomeric lattice (Figure 4D). Fluorescence recovery after photobleaching (FRAP) experiments in *unc-45* and *daf-21* RNAi-treated nematodes revealed that this deposited myosin protein was non-diffusible (Figure 4E, 4F), implying that mislocalized cytoplasmic myosin accumulates upon reduction of either DAF-21 or UNC-45 levels. As extensive disruption of the sarcomeric lattice was neither observed upon *daf-21* RNAi nor upon *unc-45* RNAi treatment, it is tempting to speculate that the fluorescent myosin patches are connected to the loss of motility (Table 1).

### Muscular expressed DAF-21 is associated with the M-line and I-band of the muscle structure

**Figure 5:**
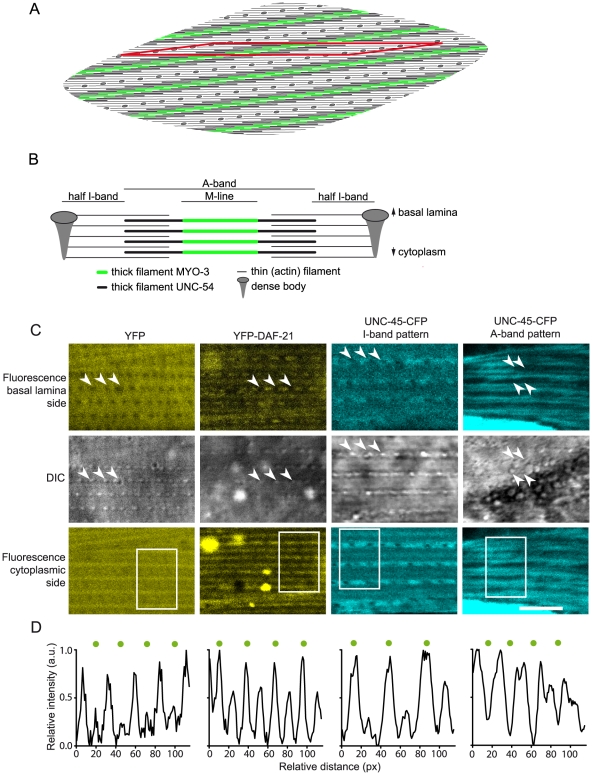
YFP-DAF-21 and UNC-45-CFP show a specific distribution within the muscular ultrastructure. A. A top-view schema of a body wall muscle cell of *C. elegans*. The illustration clearly highlights the oblique character of the striation, as sarcomeres run in an angle of 6° in relation to the macroscopic striation. The MYO3-rich M-line of thick filaments is highlighted in green. The red rhomboid is one sarcomere wide. B. A section along the long edge of the rhomboid in (A) reveals the sarcomere structure more clearly. Between two rows of dense bodies the actin filaments span into the I- and A-band. Within the A-band the thick filaments are found which consist of UNC-54 (black) and MYO-3 (green) in their center, which is the M-line. The myosin attachment sites in the M-line have been omitted for clarity. C. Confocal image stacks of muscle cells of YFP (strain AM134), YFP-DAF-21 and UNC-45-CFP expressing nematodes were recorded. Fluorescence micrographs of optical sections through the sarcomere lattice close to the basal lamina are presented together with simultaneously recorded DIC images. The dense bodies are indicated by arrows in the DIC images and the corresponding positions are marked in the fluorescence image. Below, fluorescence micrographs of optical sections closer to the muscle cell body taken from the same image stacks are presented. The scale bar represents 5 µm. D. The striation patterns of the white frames in the micrographs of panel (C) were analyzed using the plot profile module of ImageJ. Measured intensities (bottom to top in the corresponding micrograph) are presented as plots of normalized pixel intensity versus pixel position. The peaks corresponding to the I-band are marked (green dots) in order to allow comparison of relative fluorescence distribution.

The nematode muscle ultrastructure is related to that of higher metazoan systems, but muscle fibers formed by the in line connection of sarcomeric units do not exist. Nematode body wall muscle cells contain a limited number of parallel M-lines, which are formed by the middle part of each myosin filament and can be visualized via the MYO-3-GFP construct utilized before. Between two M-lines are the I-bands, which contain the dense bodies as actin attachment sites and thus serve a function analogous to the Z-line of mammalian sarcomeres. Contrarily to mammalian sarcomeres, the actin and myosin filaments are not arranged perpendicular to the I-band, but oblique in a angle of 5–7° [41], [42] (Figure 5A, 5B).

As DAF-21 seems to be required for the correct localization of myosin into the sarcomeric lattice, we analyzed the subcellular localization of DAF-21 in muscles of living nematodes. We generated a YFP-fusion construct of DAF-21 by inserting YFP into the long, flexible linker region, which connects the N-terminal and the middle domain of DAF-21 (YFP-DAF-21). Both, the N-terminus and the C-terminus of Hsp90 are known to be important for its enzymatic activity and cofactor interaction, while the 60 amino acid linker region had previously been shown to be not essential for proper function [43]–[45]. We tested the functionality of the YFP-fused DAF-21 protein using a recombinantly expressed and purified version of it. We found the chaperone activity of DAF-21 to be fully maintained in a citrate synthase aggregation assay (Figure S4). The ATPase activity of the protein was reduced compared to wt DAF-21, but a stimulation of the activity by CeAha1 could still be observed (Figure S5). Furthermore, binding of the TPR-containing cofactors PPH-5 and most importantly the interaction with an N-terminal fragment of UNC-45 (amino acids 1–461) is possible (Figure S6, S7). In addition, these ultracentrifugation experiments also revealed that YFP-DAF-21 can form heterodimers via its C-terminal dimerization site, suggesting that the formation of heterodimers with endogenously expressed DAF-21 would be possible (Figure S8). Thus, the functionality of DAF-21 is maintained in this YFP fusion protein.

We used the well-established *unc-54* promoter to target YFP-DAF-21 to the body wall muscle cells. Interestingly, YFP-DAF-21 was not homogeneously localized within body wall muscle cells. Instead, it exhibited a striated pattern, closely matching the striation of muscle cells. To determine at what band of the striation YFP-DAF-21 is enriched, we recorded Z-stacks of whole cells and compared confocal fluorescence micrographs to simultaneously recorded DIC micrographs, using the clearly visible dense bodies as landmarks for the localization of the I-band and consequently the other bands of the myofibrillar lattice [41]. The dense bodies were clearly visible in the DIC channel (Figure 5C). Overlaying the DIC image and the simultaneously recorded fluorescence optical section through the lattice in a plane very close to the basal lamina revealed the fluorescence being localized to the space between the dense bodies, clearly excluded from those and the remainder of the I-band (Figure 5C). The continuous line of DAF-21-localization between two rows of dense bodies correlates well to the broad A-band, probably even specifically to the M-line, as localization is sharply restricted to the very middle of the A-band. Comparing several cells with different expression levels, it appears that the localization to the M-line can only be observed if expression levels are high, while in cells expressing lower levels of YFP-DAF-21, the fusion protein only localizes to the I-band.

To understand, whether this pattern of localization is characteristic for DAF21, we compared it to that of the nematode strain AM134, which expresses YFP in body wall muscle cells (Figure 5C). Here as well, we could observe striation and the exclusion of fluorescence from the dense bodies. However, the protein is uniformly distributed throughout the fibrillar lattice. The comparison of the YFP-DAF-21 patterns and that of YFP in an optical sectioning plane closer to the muscle cell body (Figure 5C) revealed that YFP-DAF-21 was still mainly localized to the I-band, with the M-line localization being visible. Quantification of the bands clearly illustrates that within the repetitive structure of the muscle fiber, the two bands show enrichment of fluorescent protein (Figure 5D). In the GFP-expressing AM134 strain in a similar sectioning plane, the M-line was slightly enriched in fluorescence but still exhibited a high background of homogeneous distribution (Figure 5C and D). Therefore, a specific enrichment of YFP-DAF-21 in the I-band can be observed, while both, YFP only and YFP-DAF-21 apparently can accesses the M-line of the muscular structure.

To compare the localization of DAF-21 and UNC-45, we generated nematodes, which express UNC-45-CFP in body-wall muscles. We determined the protein's localization as described above (Figure 5C). Interestingly, the muscular bands containing DAF-21 also contained UNC-45-CFP in most cells (I-band pattern). But in contrast to DAF-21, we observed localization in two distinct patterns. About 20% of the cells showed UNC-45 specifically in those areas, where DAF-21 had been excluded from. In these cells, a clear localization of UNC-45-CFP to the A-bands is visible, while the thin M-line between them is excluded (A-band pattern). Thus it appears that the accessibility of the two proteins to the muscular ultrastructure overlaps mostly in the region of the I-bands and M-line, while other parts of the myofibrillar lattice appear only accessible to UNC-45.

### YFP-DAF-21 is dynamically localized to the I-band of the muscles, while UNC-45 can stably bind to the A-band

**Figure 6:**
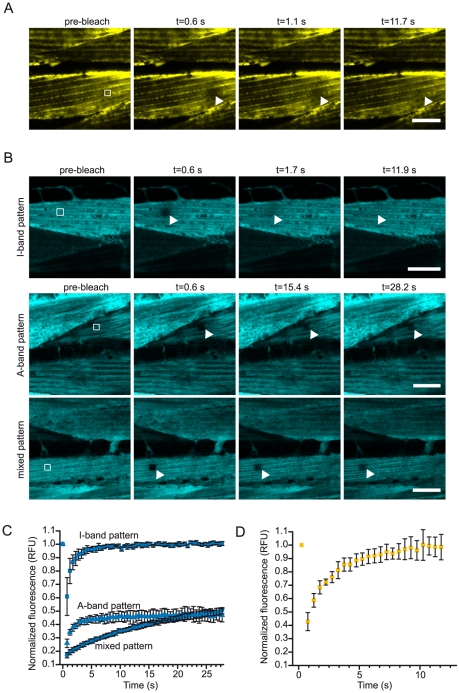
YFP- DAF-21 and UNC-45-CFP differ in their dynamic properties. A. Nematodes expressing YFP-DAF-21 were subjected to photobleaching. Images before bleaching and during recovery at the indicated time points are shown. The bleached area is highlighted by a white frame. The scale bar represents 10 µm for all micrographs in the panel. B. FRAP analysis was also performed on UNC-45-CFP expressing nematodes. UNC-45-CFP protein exhibiting the I-band pattern recovered immediately after photobleaching (upper panel), while protein localizing to the A-band did not recover within observation time (middle panel). A mixed pattern (lower panel) could also be observed, where recovery was slow. The scale bar represents 10 µm for all micrographs in the panel. C. Kinetic evaluation of the recovery phase of UNC-45 populations. Independent cells of the indicated type were evaluated averaging triplicate FRAP experiments. The error bars represent the standard deviation. Full recovery of the I-band pattern could be observed, while the mixed pattern and the A-band pattern recover dramatically slower and to lower intensities implying that in these experiments stably attached protein was monitored. D. Kinetic evaluation of YFP-DAF-21 recovery. All FRAP experiments with YFP-DAF-21 were characterized by a fast and complete recovery phase.

We used FRAP to determine, whether the observed localization is a stable association or whether it is dynamic. We bleached YFP-DAF-21. The loss of fluorescence was barely observable, indicating a very rapid recovery of fluorescence by freely diffusing YFP-DAF-21 (Figure 6A). We therefore conclude that the detectable amount of the DAF-21 is not stably attached to the muscular structure, but appears to contribute to muscular stability as a transiently associated factor.

We also analyzed UNC-45-CFP by FRAP. Using cells, which showed the I-band pattern, we observed that the protein localized to these structures is also freely diffusible (Figure 6B, upper row). We also used FRAP analysis in the cells, which showed UNC-45-CFP localization to the A-bands. Surprisingly, recovery was very slow and even after five minutes, the bleached area had not recovered its fluorescence fully (Figure 6B, middle row). Analyzing more cells, also intermediate expression patterns were observed (Figure 6B, lower row). This also becomes evident in the quantitative analysis of the recovery phase, where a mixture of different UNC-45-CFP populations can be observed (Figure 6C). Instead, all bleaching experiments with DAF-21-YFP resulted in fast recovery phases (Figure 6D). Thus, UNC-45 appears stably associated to the A-band, while the protein localized to the I-band is – like DAF-21 – freely diffusible. These observations hint to two distinct populations of UNC-45 in muscle cells and might be explained by a variable number of accessible UNC-45 binding sites at the A-band, which upon saturation allow the accumulation of a freely diffusible UNC-45 population.

## Discussion

Hsp90 is known to be an essential protein in many organisms, including *S. cerevisiae*, *C. elegans*, *D. melanogaster* and vertebrates [11], [16], [17], [26], [46]. Here, we address the functions of the Hsp90-homolog DAF-21 in the nematode *C. elegans*, where we and others had recently found an involvement in the development of the gonad [13], [15], [47]. Beyond this, DAF-21 apparently plays a crucial role in maintaining the nematode's muscular structure and function. We find that a DAF-21 mutation, which reduces the enzymatic activity of Hsp90, but retains most of its other functions, results in motility defects and other phenotypic traits related to dauer development, chemotaxis and thermotaxis [26], [48], [49].

### DAF-21 and UNC-45 are required for proper motility and myosin localization

**Figure 7:**
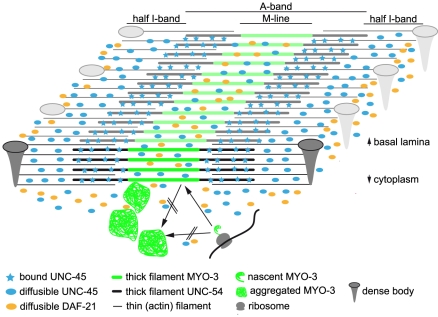
A model for the sarcomeric distribution and mode of action of DAF-21 and UNC-45. A three-dimensional schema of a sarcomere. The oblique character of striation has been ignored for reasons of clarity. Two dense bodies frame the sarcomere, consisting of actin thin filaments on both sides and myosin thick filaments in the central region. The green area of the thick filaments corresponds to the MYO-3 rich M-line, as observed in the reporter strain RW1596. UNC-45 protein stably associated to the A-band is depicted as light blue stars. Please note the differential distribution of soluble factors, as DAF-21 (yellow ellipsoids) is mainly found in the space between dense bodies, whereas soluble UNC-45 (light blue ellipsoids) distribution reaches further into the central area of the sarcomere. Cytoplasmic aggregates of MYO-3 are depicted as green tangles, which may result from newly synthesized MYO-3 or MYO-3 derived from the sarcomeric lattice.

According to our data, the knockdown of *daf-21* leads to motility defects, the muscular induction of the heat shock response and the incorrect deposition of MYO-3 into aggregate-like structures close to the myofibrillar lattice (Figure 7). The origin of the aggregated MYO-3 protein is not known: as during the RNA interference experiments all nematodes pass through the larval stages to become adult, the muscular ultrastructure expands by incorporation of new myosin subunits. It however cannot be distinguished whether the aggregate-like structures are composed of newly synthesized myosin about to be assembled into the lattice or whether aggregated MYO-3-GFP originates from the existing ultrastructure. It is clear that the aggregate-containing parts of the cytosol should not contain detectable amounts of MYO-3 protein in normal muscle cells. Previous *in vitro* studies had indicated that a direct interaction of DAF-21 with its cofactor UNC-45 is required for the folding of the UNC-54 myosin head domain and its correct assembly into the myofibrillar lattice [50], [51]. Instead, no interaction was observed with the myosin MYO-3 before [50]. Thus, during the cellular turnover process of myosins, the aggregation of MYO-3 may represent the final outcome of a more complex series of misguided events in response to the loss of either DAF-21 or UNC-45.

The involvement of Hsp90 in the muscular organization had been observed in vertebrates before. Studies in zebrafish showed that Hsp90 is involved in myofibrillogenesis. The specific knockdown of Hsp90a.1 resulted in the distortion of muscle fibers [19]. The morphological homology between muscle cells of vertebrates and nematodes is limited – the striation in nematodes is not arranged perpendicular to the sarcomeric organization, but oblique [42]. Identifying Hsp90 as an important contributor to muscular maintenance in nematodes however recapitulates this finding also in lower metazoan species and may expand the possibilities to address Hsp90 functions mechanistically in another *in vivo* system. As the homolog of UNC-45 in yeast lacks the TPR-domain, which interacts with Hsp90, the function of Hsp90 in the maintenance of muscle structure may represent an evolutionarily novel utilization of Hsp90's ability to support client proteins in multicellular eukaryotes, which adds to the chaperoning activity for transcription factors and protein kinases.

### Mechanism of DAF-21 and UNC-45 involvement in muscle maintenance

The contribution of DAF-21 and UNC-45 to muscle maintenance is still not clear. In *C. elegans*, solely the involvement of the myosin binding protein UNC-45 in muscle formation has been addressed before [22], [23], [52]. In fixated nematodes it was found to be localized to the myosin containing A-band, the region of the muscle containing the myosin homolog UNC-54 [22], [23]. Complementary studies in zebrafish observed a shuttling of Unc-45 and Hsp90a.1 between Z-line and A-band in response to damage [53], [54].

In this study, using live nematodes expressing fluorescently tagged proteins, we observed differences in the subcellular localization of DAF-21 and UNC-45 which may hint to a sophisticated tempo-spatial regulation of their interaction. UNC-45, if present in abundance, is localized mainly to the I-band (Figure 7). All parts of the muscular ultrastructure appear accessible. The bulk of UNC-45 is not stably associated with any structure, as it recovers at a fast rate after photobleaching. Some cells, however, reveal that not all UNC-45 is freely diffusible. Here, UNC-45 can be seen stably associated with the A-bands. This hints to two populations of UNC-45, one freely diffusible in the cytosol and the other stably attached to myosin filaments. Interestingly, the center of the A-band appears to contain less UNC-45-CFP protein (Figure 5C). This space corresponds to the MYO-3 containing M-line and could confirm that stably attached UNC-45 localizes preferentially to the UNC-54 containing part of the A-band (Figure 7). It is interesting to note, that these two patterns in all observed cases affect the whole cell and not subcellular regions of the sarcomeric lattice. This concerted reaction in the cell could hint to a regulated event.

For DAF-21 instead, no stably associated protein could be detected (Figure 7). In cells, expressing little DAF-21, its localization was still restricted to the central I-band between the dense bodies. This localization may correlate to the Z-line localization described for vertebrate skeletal muscle cells [54]. The protein can be detected in this area and in parts of the A-band. The localization within the A-band appears specific for the thin M-line, that also contains MYO-3 and potentially is excluded from the UNC-54 containing part of the thick filament. Thus, the localization of DAF-21 seems more spatially constrained than that of UNC-45. This may be due to different affinities to or protein specific exclusion from parts of the ultrastructure. It cannot be ruled out though that YFP-DAF-21 behaves differently from endogenous DAF-21, as some properties of the chaperone may be altered by the fusion to YFP. Nevertheless, as the binding to the TPR-domain of UNC-45 and the chaperone activity appear to be functional, relevant aspects of DAF-21 functionality certainly are still supported. Based on our data, Hsp90 contributes to the maintenance of muscular integrity as a soluble, transiently associated factor, while UNC-45 can stably associate with the UNC-54 containing part of the myofibrillar lattice under yet to be defined circumstances.

## Materials and Methods

### Nematode growt and, cultivation

All *C. elegans* strains used in this study were cultivated according to standard procedures [55]. Worms were grown on nematode growth medium (NGM) agar plates seeded with *E. coli* OP50 strain at 20°C. For synchronization, worms were bleached in sodium hypochlorite solution and eggs or hatched L1 larvae were transferred to fresh NGM plates. The nematode strains N2, JT6130 (*daf*-*21(p673)*), AM134 (*rmIs126*), RW1596 (*myo-3(st386)*) and BC10293 (*sEx10293*) were obtained from the Caenorhabditis Genetics Center (CGC, Minneapolis, USA), the heat shock reporter strain containing an integrated *hsp-70::GFP* construct (*rmIs8*) was obtained from Richard I. Morimoto [28], [39].

### Lifespan and motility assays

In order to measure the adult lifespan nematodes were synchronized and grown at 20°C on NGM plates. On day 4, young adult (YA) stage nematodes from N2 and JT6130 (*daf-21(p673)*) strains were picked and transferred to new NGM plates, thereby excluding all JT6130 nematodes that entered the dauer state. Nematodes were transferred to new NGM plates every two days to separate them from their progeny. The number of living animals was scored every second day. Worms were considered dead if they did not respond to prodding with a platinum wire and when pharyngeal pumping was absent. Additionally, nematodes which harboured their progeny (bagged worms) were counted as dead.

Thrashing assays were carried out to measure the motility of the surviving nematodes. Worms were placed in a drop of M9 buffer and the number of lateral swimming movements performed per minute was counted [56]. For each time point, 15 adult hermaphrodites were subjected to thrashing assays. These assays were performed on day 4, day 7 and day 10 after hatching. In a similar manner, the effect of RNAi against *daf-21* and *unc-45* was analyzed.

### Generation of transgenic nematodes

An expression reporter construct was generated by cloning the 2.5 kb genomic sequence upstream of the *daf-21* start codon, including the codons for the first four amino acids in frame to the YFP gene (Clontech, Mountain View, USA) in the plasmid pPD95.79 using the primers GGA TTC GCG GCC GCA GAA AGT ATC TCG AAG CCA TCG GCA GTT TAT G and TGA CGC TAG CGT TCT CGG ACA TGG TTC TGG AAA AAT. In order to analyze the muscular association of DAF-21 a YFP-DAF-21 fusion construct was generated. As the enzymatically important N-terminus [57] and the C-terminal interaction with TPR-domain containing cofactors [58], like UNC-45, should not be disturbed, the YFP moiety was integrated into the flexible linker region between the N-terminal and middle domain of DAF-21 (YFP-DAF-21). This linker region is fully dispensable in yeast [43]–[45] and varies in length between 60 and 90 amino acids in eukaryotes. This construct was inserted into the plasmid pPD30.38, leading to muscular expression under the control of the *unc-54* promoter. Also, a muscular expressed UNC-45 fusion protein was constructed, which contains CFP at the C-terminus. Microinjections were performed according to standard procedures [59] by injecting a solution of 100 µg/ml plasmid DNA into the distal gonads of YA worms. Injections were performed on a Zeiss Axiovert 200 microscope (Zeiss Microimaging, Jena, Germany) equipped with an injection device (Eppendorf, Hamburg, Germany). Existence of fluorescent F1 progeny was observed using a Leica MZ-16FA (Leica Microsystems, Wetzlar, Germany) fluorescence microscope. In order to obtain stable lines, fluorescent offspring were transferred to new NGM plates.

### Microscopy and confocal microscopy

Fluorescence micrographs were either generated, using a Leica MZ-16FA (Leica Microsystems, Wetzlar, Germany) or a Zeiss Axiovert 200 microscope equipped with a Hamamatsu C4742-95 camera (Hamamatsu, Herrsching, Germany). RNAi motility phenotypes were scored using a Zeiss Stemi stereo microscope (Zeiss Microimaging, Jena, Germany). DAF-21 and UNC-45 association to the myofibrillar lattice was studied by confocal microscopy using a Leica SP5 laser scanning microscope. YFP and CFP were excited, using the 514 nm and 458nmlines of an Argon laser, respectively. For imaging, 4% of the total laser power was deployed. Gain and offset were adjusted to fully exploit the dynamic range of the photomultipliers. Fluorescence recovery after photobleaching (FRAP) in life animals was carried out using the same laser scanning microscope. A defined area was scanned at imaging speed in zoom-in mode at 70% of the full laser power. Recovery was tracked until a stable plateau was reached. Image manipulations, if necessary, were linear adaptation of contrast and brightness and were performed using ImageJ. For intensity profile plots the appropriate plugins of ImageJ were used [60].

### RNA interference experiments

RNAi experiments were performed by feeding dsRNA-expressing *E. coli* to the nematodes according to standard procedures [61], [62]. The RNAi constructs directed against *daf-21*, *unc-45* and the empty control vector L4440 (designated ‘control RNAi’ throughout the manuscript) were described before [14], [28] and sequenced prior to performing the assays. Individual colonies of dsRNA expressing *E. coli* HT115 (DE3) were grown in LB_Amp,Tet_ medium and dsRNA expression was induced by the addition of IPTG to a final concentration of 1 mM. After 4 hours, bacteria were placed on NGM agar plates containing 50 µg/ml ampicillin, 6 µg/ml tetracycline, 1 mM IPTG and 5 µg/ml cholesterol. L1 larvae were grown on these plates at 20°C and scored at the indicated age.

### Quantitative Real-Time PCR experiments

To analyze gene activation in response to heat shock on a transcriptional level, Quantitative Real-Time PCR (QRT-PCR) experiments were performed. For induction of the heat shock response, N2 nematodes of a mixed healthy population were heat shocked at 35°C for 1 h in a water bath, on plates sealed in plastic bags. After heat treatment, worms were washed off their plates with M9 medium and bacteria were removed by several washing steps. To isolate RNA the RNeasy Mini Kit (Qiagen, Hilden, Germany) was used. Worms were disrupted in buffer RLT supplemented with β-mercaptoethanol using glass beads in a MM400 swing mill (Retsch, Haan, Germany) at 30 Hz three times for 2 min. The lysate was applied to spin columns and the manufacturer's protocol was followed. A total amount of 10 µg of RNA was subjected to reverse transcription with random hexamer primers using ALV reverse transcriptase (Promega, Madison). QRT-PCR was carried out according to the manufacturer's instructions, using the GoTaq qPCR MasterMix (Promega, Madison, USA) in a MX3000P QPCR System (Agilent Technologies, La Jolla, USA). Primer pairs were designed to amplify a 200 to 250 bp segment of spliced target gene mRNA by using at least one primer per pair that binds on exon-exon junctions. Specificity of the RT-PCR reaction also was confirmed on 1% agarose gels. The used primer pairs were for *act-1* fwd: AAT CCA AGA GAG GTA TCC TTA, rev: GAT GGC GAC ATA CAT GGC T; *pgk-1* fwd: TTT GAT CCG TGT TGA CTT CAA T, rev: GAA GAG AAC ATC TTT TTT CAA GA; *daf-21* fwd: AAG ATG AGG AGG CTG TCG A, rev: CAT TGG ACA AGC TCT TGT AGA; *hsp-1* fwd: CAC TGT TTT CGA TGC CAA AC, rev: TCT CCT TCA TCT TCA GCA AAA; *hsp-70* fwd: TTT CAA TGG GAA GGA CCT CAA, rev: TTG GAA GCT TTG GCA GGA ATT; *hsp-16.11* fwd: TCT GAA TCT TCT GAG ATT GTT AA, rev: CTT CTG AAA GAT TTG AAG CAA CT. The log 2 fold change of the genes of interest upon heat shock was calculated versus *act-1* and *pgk-1* as normalizers and averaged for four separate biological experiments.

### Expression cloning and protein purification

Expression plasmids for CeAha1 (C01G10.8), STI-1, PPH-5 and Cep23 (ZC395.10) and Unc-45N (aa1–461) were generated by PCR from cDNA-containing RNAi clones (BioCat, Heidelberg, Germany). The E292K variant and the C-terminal fragment of DAF-21 (aa524–702) were generated using the *daf-21* cDNA as a template [14]. PCR-products were inserted into the pET28b expression plasmid (Merck, Darmstadt, Germany) and verified by DNA sequencing (GATC Biotech, Konstanz, Germany). YFP-DAF-21 cDNA was as well subcloned into the pET28b expression vector for recombinant protein expression. Proteins were expressed in BL21-CodonPlus (DE3)-RIL bacteria (Stratagene, LaJolla, USA). Bacterial cultures were grown to an OD_600_ of 0.8 and induced with 1 mM IPTG. Cells were disrupted using a cell disruption machine (IUL Instruments, Freiburg, Germany) and the lysate was loaded onto a HisTrap FF column (GE Healthcare, Freiburg, Germany). Protein was eluted in a buffer containing 400 mM imidazole. Eluted protein was dialyzed and applied to a ResourceQ anion exchange column (GE Healthcare, Freiburg, Germany). Finally, proteins were subjected to size exclusion chromatography on Superdex 75 or 200 PrepGrade gel filtration columns (GE Healthcare, Freiburg, Germany). Protein purity was assessed by SDS-PAGE and the molecular mass was determined by a MALDI-TOF/TOF mass spectrometer (Bruker, Bremen, Germany). Proteins were frozen in liquid nitrogen in 40 mM HEPES/KOH, pH 7.5, 20 mM KCl, 0.2 mM EDTA, 1 mM DTT.

### ATPase measurements

The ATPase activity of DAF-21, E292K-DAF-21 and YFP-DAF-21 was determined using an ATP regenerating system as described previously [6], [63]. Measurements were carried out using a premix consisting of phosphoenolpyruvate, NADH, LDH and pyruvate kinase (Roche Diagnostics, Penzberg, Germany) in a buffer containing 40 mM HEPES/KOH, pH 7.5, 5 mM MgCl_2_ and 80 mM KCl. Assays containing CeAha1 were performed in low salt buffer (40 mM HEPES/KOH, pH 7.5, 20 mM KCl, 5 mM MgCl_2_) to increase the affinity as described before [64]. The concentration of the DAF-21 variants was 4 µM. The temperature of the experiments was varied by using a Varian Cary50 UV/Vis spectrophotometer attached to a thermostat. ATPase activites were suppressed by radicicol in order to determine the background activity. This background activity, which was only observable in the case of YFP-DAF-21, was then subtracted.

### Urea induced unfolding transitions

Urea induced unfolding transitions were performed by diluting DAF-21 and E292K-DAF-21 to a concentration of 500 nM into storage buffer supplied with the indicated molarity of urea. The samples were incubated at room temperature for 16 h until the unfolding equilibrium was reached. Emission spectra at an excitation wavelength of 280 nm were recorded.

### Citrate synthase aggregation assays

To check the chaperone activity of DAF-21, E292K-DAF-21 and YFP-DAF-21, citrate synthase aggregation assays were performed. Citrate synthase was prepared as described [65]. Different concentrations of DAF-21 and variants were incubated with 500 nM citrate synthase in 40 mM HEPES, pH 7.5 at 43°C and protein aggregation was followed using a spectrophotometer at 360 nm.

### Analytical ultracentrifugation (AUC)

Binding of cofactors to DAF-21 and to the indicated variants as well as heterodimer formation was analyzed by analytical ultracentrifugation with fluroescence detection [66]. Experiments were performed in a Beckman ProteomeLab XL-A analytical ultracentrifuge (Beckman, Brea, USA) equipped with an Aviv AU-FDS detector (Aviv Biomedical, Lakewood, USA). The cochaperones were labelled with 5-carboxyfluorescein N-succinimidyl ester (Invitrogen, LaJolla, USA) at lysine residues. They were subjected to ultracentrifugation in the presence and absence of DAF-21, its variants and nucleotides as indicated in the figure legends. Centrifugation was performed at 20 to 22°C and 42.000 r.p.m for 12 hours and scans were recorded every 90 seconds. The fluorescently labelled cochaperones sedimented with the same characteristics as the unlabelled proteins. Data analysis was performed using a dc/dt approach according to Stafford [67]. The dc/dt plots were fit to bi-gaussian functions in order to obtain the s values of the respective species as described previously [68].

AUC also was performed to determine the binding of cofactors and the C-terminal fragment of DAF-21 to YFP-DAF-21. Here, the fluorescence of YFP was used to detect the sedimentation process. For all AUC experiments using fluorescence detection, the concentration of the labelled species was 300 nM, the concentration of binders 6 µM. Binding of UNC-45N (10 µM) to unlabelled DAF-21 (5 µM) was followed by interference optics. Data analysis was carried out as described above.

## Supporting Information

Figure S1 **DAF-21 and E292K-DAF-21 are comparably stable upon chemical denaturation.** Urea induced unfolding transitions of DAF-21 (filled circles) and E292K-DAF-21 (open circles) were performed as described in Materials and Methods. The transitions show the intrinsic fluorescence measured at 315 nm in dependence of the urea concentration. Both proteins unfold in a highly cooperative fashion at a urea concentration of 4.9 M.
(EPS)

Figure S2 **The position of E292 in the crystal structure of Hsp90.** A cartoon of the structure of yeast Hsp90, which is highly homologous to DAF-21. One protomer of the dimer is depicted in blue. Within the other protomer the N- and C-terminal domains are colored in green and the middle domain in gold. The highly conserved E292 residue is highlighted in red. The figure was adapted from PDB structure 2CG9 [69].
(EPS)

Figure S3 **DAF-21 and E292K-DAF-21 are active chaperones.** Thermally denatured citrate synthase aggregation (black boxes) is inhibited by the addition of 0.1 µM DAF-21(grey circles) and E292K-DAF-21 (green circles). Upon the addition of 0.5 µM DAF-21 (grey triangles) or E292K-DAF-21 (green triangles), aggregation of citrate synthase is fully suppressed.
(EPS)

Figure S4 **DAF-21 and YFP-DAF-21 are active chaperones.** Thermally denatured citrate synthase aggregation (black boxes) is inhibited by the addition of 0.1 µM DAF-21(grey circles) and YFP-DAF-21 (yellow circles). Upon the addition of 0.5 µM DAF-21 (grey triangles) or YFP-DAF-21 (yellow triangles), aggregation of citrate synthase is fully suppressed.
(EPS)

Figure S5 **The hydrolysis rate of YFP-DAF-21 is reduced, but can be stimulated by CeAha1.** The hydrolysis rate of YFP-DAF-21 is reduced in comparison to DAF-21. Nevertheless, upon the addition of 6 µM CeAha1, the ATPase of YFP-DAF-21 can be stimulated. The ATPase assays were performed at 25°C in low salt buffer as described in the Materials and Methods section.
(EPS)

Figure S6 **YFP-DAF-21 can bind TPR domain containing cofactors.** Following the sedimentation of YFP-DAF-21 in AUC experiments by fluorescence detection, the capability to bind to TPR-domain containing proteins is confirmed. Despite the YFP insertion, YFP-DAF-21 binds UNC-45N (blue circles), PPH-5 (green circles) and STI-1 (red circles), as indicated by a shift to higher sedimentation coefficients.
(EPS)

Figure S7 **A TPR-domain containing fragment of UNC-45 binds to DAF-21.** The interaction of UNC-45N and DAF-21 is confirmed by AUC. UNC-45N (grey circles) and DAF-21 (black circles) were sedimented alone or as a mixture (blue circles). UNC-45N alone has a sedimentation coefficient of 3.8 S, DAF-21 of 5.9 S. Adding the cofactor in a twofold molar excess to DAF-21, the UNC-45N peak at 3.8 S loses intensity and the DAF-21 peak shifts – due to binding – to a higher s value at 6.7 S. The sedimentation of the proteins was followed by interference optics.
(EPS)

Figure S8 **YFP-DAF-21 can form heterodimers with a C-terminal fragment of DAF-21.** Fluorescence detection AUC confirms heterodimer formation of a C-terminal fragment of DAF-21 and YFP-DAF-21. YFP-DAF-21 has a sedimentation coefficient of 7.3 S (yellow circles). By adding the C-terminal fragment of DAF-1 (aa524–702), the sedimentation coefficient shifts to 6.0 S implying heterodimer formation and a consequent loss of molecular mass.
(EPS)
